# Understanding Patient-Reported Offenses in Electronic Health Records: Cross-Sectional Mixed Methods Survey

**DOI:** 10.2196/86178

**Published:** 2026-05-14

**Authors:** Saija Simola, Sari Kujala, Åsa Cajander, Anna Kharko, Bridget Kane, Bo Wang, Rose-Mharie Åhlfeldt, Maria Hägglund

**Affiliations:** 1 Department of Computer Science, Aalto University, Konemiehentie 2, Espoo, 02150, Finland, 358 509118203; 2 Department of Information Technology, Uppsala University, Uppsala, Sweden; 3 Department of Women’s and Children’s Health, Participatory eHealth and Health Data Research Group, Uppsala University, Uppsala, Sweden; 4 Centre for Primary Care and Health Services Research, University of Manchester, Manchester, United Kingdom; 5 Business School, Karlstad University, Karlstad, Sweden; 6 Norwegian Centre for E-health Research, University Hospital of North Norway, Tromsø, Norway; 7 School of Informatics, University of Skövde, Skövde, Sweden; 8 Uppsala University Hospital, Uppsala, Sweden

**Keywords:** electronic health record, online record access, patient portal, user groups, offensive, national survey, open notes, patient experiences, patient-accessible electronic health records

## Abstract

**Background:**

Patients’ access to their electronic health record (EHR) supports their participation and satisfaction with care. Despite the benefits, some patients have been upset after reading their EHR. Additionally, health care professionals are concerned that patients, particularly those with mental health conditions, may be offended, and they have expressed a need for further guidelines on how to write EHRs. Experiences among various patient groups are essential to support the relationship between patients and professionals. However, prior studies have often focused on single patient groups or specific clinical contexts, leaving a limited understanding of differences across multiple patient groups.

**Objective:**

This study aimed to determine whether certain patient groups are more likely to feel offended while reading their EHRs and which information is perceived as offensive and to provide a comparison across multiple patient groups using a mixed methods approach.

**Methods:**

A cross-sectional survey was conducted via the Finnish national patient portal using a web-based patient survey, adopting a mixed methods approach. The survey included multiple-choice and open-ended questions. The total sample comprised 4681 respondents. The survey respondents were placed into 4 patient groups: those who had received care for mental health, cancer, or other conditions and those who had received no care. Associations between the type of care and patients who felt offended were estimated using multivariate binary logistic regression. Inductive content analysis (n=502) was conducted to identify information perceived as offensive in the EHR.

**Results:**

The patients who had received mental health care (166/654, 25.4%) or cancer and mental health care (9/39, 23.1%) were more likely to be offended by information in their EHR compared to the other groups (cancer care: 37/375, 9.9%; other conditions care: 383/3316, 11.6%; no care: 22/206, 10.7%; other conditions care: odds ratio 0.37, 95% CI 0.29‐0.46; *P*<.001; model A). Additionally, female patients, those with bad or very bad health conditions, and patients with bachelor’s or master’s degrees were significantly more likely to feel offended. Errors, the health care professionals’ disrespectful language, and perceived unnecessary information were the most frequently mentioned reasons for being offended. Patients with mental health care reported more often that unnecessary information and professionals’ opinions and word choices were experienced as offensive compared to other patients.

**Conclusions:**

This study contributes new knowledge by identifying differences across multiple patient groups. Although a minority of patients felt offended by their EHR, health care professionals should consider that some patients, particularly those who have received mental health care or cancer and mental health care, may be offended by specific information or word choices in their EHRs. To address this, health care professionals should receive education on how to write their notes in a neutral tone and avoid potentially offensive topics. Improving the quality of EHRs could strengthen the relationship between patients and professionals.

## Introduction

### Background

Increasingly, countries are offering patients access to their electronic health records (EHRs) because it benefits both patients and the health care system [1]. The Northern European countries, including Finland, have a long history of planning national EHRs to support patients’ autonomy and participation in alliance with health care professionals’ access to the records [2]. Available through patient portals, EHRs include medical information, such as appointment notes, diagnoses, and laboratory test results [3,4]. Patients’ access to their EHRs can improve communication between patients and health care professionals [5,6], as well as patients’ participation [7,8], satisfaction [9], trust [10], and engagement with care [11,12]. Additionally, patients’ access to their EHRs can encourage them to take their medicines as prescribed [13], support their recall [7,9,11,14], and enhance their understanding of care [8,12].

While patients’ access to their EHR appears to be beneficial to patients in general, differences between patient groups have been identified. Some patients with mental health conditions have reported experiencing these benefits more frequently than those without mental health issues [11,15-17]. Health care professionals have found that using EHRs might help patients better understand their mental health conditions, including their diagnoses, care plans, medications, and treatments [15,18]. Patients’ access to their EHR is even more likely to encourage them with serious mental health conditions to take their medications as prescribed compared to patients without mental health diagnoses [18]. In addition, patients with cancer have reported experiencing less uncertainty or anxiety [19], and patients with complex chronic conditions have shown a decreased likelihood of visiting the emergency room and having hospital stays if they have access to the EHR [20].

Access to the EHR can also cause negative emotions in patients, including those with mental health conditions, and EHR content that upsets, offends, or judges them can deter them from reading their EHR [16,21,22]. Increased anxiety has been reported among some oncologic patients after reading their EHR [19,23]. Around 11% to 10% of patients, including oncologic patients, who read their EHR feel offended [13,22-24]. However, patients who had received mental health care have been more often offended than other patient groups [25,26]. Feeling offended by the EHR content is also associated with poorer reported perceived usability of the patient portal [24]. On the other hand, patients have indicated a willingness to access their EHRs despite potential challenges arising from their records, such as feeling offended by the content [12].

Such potential negative consequences are commonly cited as reasons why some health care professionals resist patients’ access to their EHRs. Some professionals expect patients to worry more [27] or potentially misunderstand the EHR information [28]. Health care professionals are also concerned about the potential worsening of their relationship with their patients [28-32], particularly if professionals, for example, raise questions related to sensitive topics such as gender identity [33]. This introduces a dilemma because health care professionals have a medicolegal obligation to include certain information in the EHR, even if it may worry or upset the patient [10,31,34].

Due to the potential for offense, some health care professionals may not be willing to share sensitive topics or certain parts of the EHR, or they may even choose not to share records with patients at all [34-36]. This is particularly true for mental health records [9,37]. In addition, professionals have described negative consequences after patients read about their mental health, such as decreased engagement in their care, worsened relationships [9] and trust [9,17,38], and disagreements about the EHR content [9,39-41]. Moreover, psychiatrists are more concerned than nonpsychiatrists about the possibility of patients feeling offended [42]. Health care professionals also worry about patients with mental health issues facing information in their EHR that could cause negative emotional responses without the support of professionals [38].

On the other hand, there is less previous research relating to some medical specialties, such as oncology [43]. This survey study focuses on patients accessing EHR via the national patient portal. The Finnish national patient portal, My Kanta, was launched in May 2010 [44]. In addition to a patient-accessible EHR, all Finnish citizens can use various functionalities in My Kanta, such as renewing their electronic prescriptions, acting on behalf of their children, or recording their organ donation testaments and living wills [45]. Nowadays, in a cohort study, 82% of all Finnish adults have signed in to My Kanta at least once [45].

### Aims and Research Questions

To minimize and prevent detrimental impacts from patient access to their EHR, it is important to explore the kinds of information that patients experience as offensive. Moreover, health care professionals have expressed a need for further guidance on writing EHR notes and on content that may elicit negative emotions in patients [46]. Liu [47] has stated that there is no adequate philosophical theory of offense. However, based on the thematic analysis of patients’ experiences collected through a survey, Fernández et al [22] suggest that feeling offended includes experiences where EHR contained errors, surprises, labeling, and disrespectful language.

This study allowed patients to define “offense” themselves rather than selecting from predefined options. This approach avoids leading respondents and captures their own perspectives. Thus, this study aims to examine whether certain patient groups are more likely to perceive information in their EHRs as offensive. To complement previous studies, this study focuses on multiple patient groups based on the type of care they receive. Additionally, this study aims to identify which types of information patients experienced as offensive.

Our research questions were as follows: (1) Which user groups are more likely to perceive information in their EHR as offensive? (2) Which information do users experience as offensive in their EHR?

## Methods

A web-based survey was created to explore patients’ experiences with patient-accessible EHRs via a national patient portal, My Kanta, following the CHERRIES (Checklist for Reporting Results of Internet E-Surveys) [48] (Checklist 1).

### Research Design Overview

In this study, a mixed methods approach with a convergent design [49] was used, including the survey. The mixed methods approach enabled us to obtain a large sample size and to examine the differences between patient groups using the quantitative method. The qualitative responses provided a deeper understanding of the offenses experienced in the EHR. The qualitative data collection avoided the use of predefined options to avoid influencing respondents when exploring whether they had felt offended by something they had read.

This study utilized an anonymous questionnaire combining multiple-choice quantitative and open-ended qualitative questions (Multimedia Appendix 1) to collect parallel data and adopt a connecting approach [49]. The analysis was performed after all data had been collected. Because mental health and cancer are common and increasingly prevalent conditions [50-53], and patients with these conditions are also seen as vulnerable [37,54-56], we focused on patients who had received care for mental health conditions or cancer in our analysis, distinguishing them from patients who had received care for any other condition or no care in the last 24 months.

The quantitative questions included demographic information (age, gender, self-rated overall health, education level, employment, and whether they had health care professional education), experience with health care (having received care in the past 24 months for mental health, cancer, other conditions, or not having received care), and experience of feeling offended when reading their EHR. The respondents were asked to provide additional details about perceived offensive information in the EHR through the open-ended question, “Have you ever felt offended by something you read? If yes, please explain” (Multimedia Appendix 1).

### Data Collection and Sample Size

The web-based survey administered in Finland was part of the NORDeHEALTH 2022 Patient Survey [57]. The responses were collected from patients logged in to the national patient portal between January 24, 2022, and February 14, 2022, with the option of choosing between the official languages of Finland: Finnish and Swedish. In total, 4719 responses were collected in The NORDeHEALTH 2022. Patient Survey design and data collection have been described in more detail by Hägglund et al [57].

### Inclusion and Exclusion

**Figure 1.**
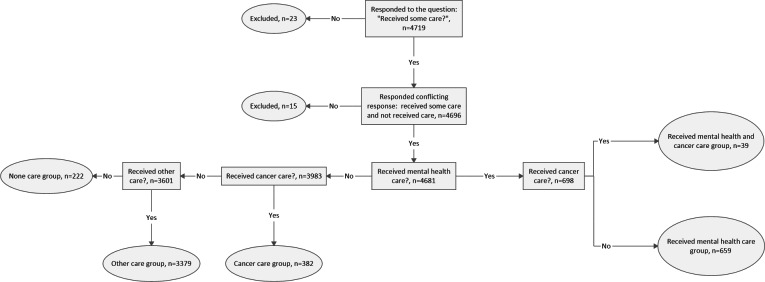
Exclusion and inclusion criteria for patient groups.

The respondents were able to select multiple groups representing the types of care they had received over the past 24 months. Thus, the patients were categorized into groups based on whether they had received mental health care, cancer care, or both. The remaining respondents were categorized into the groups “Care for other conditions” or “No care” (Table 1). Respondents who had selected both mental health care and cancer care were grouped into a separate category to avoid overlap and enable deeper analysis (Figure 1). Respondents who reported conflicting responses about receiving some care and none (15/4719, 0.3%) were excluded from the analysis. The patients who did not respond to these questions (23/4719, 0.5%) were also excluded from the analysis, resulting in a final sample of N=4681 (Figure 1).

**Table 1.** Background information categorized by a patient group.

Background information	Mental health care (n=659), n (%)	Cancer care (n=382), n (%)	Mental health and cancer care (n=39), n (%)	Other conditions care (n=3379), n (%)	No care (n=222), n (%)	Total (N=4681), n (%)
Health condition						
Very good	11 (1.7)	5 (1.3)	2 (5.1)	133 (3.9)	34 (15.3)	185 (4)
Good	145 (22)	105 (27.5)	10 (25.6)	1200 (35.5)	103 (46.4)	1563 (33.4)
Fair	308 (46.7)	195 (51)	13 (33.3)	1637 (48.4)	63 (28.4)	2216 (47.3)
Bad	135 (20.5)	54 (14.1)	12 (30.8)	322 (9.5)	14 (6.3)	537 (11.5)
Very bad	38 (5.8)	16 (4.2)	1 (2.6)	52 (1.5)	5 (2.3)	112 (2.4)
I don’t know/I don’t want to answer or no response	22 (3.3)	7 (1.8)	1 (2.6)	35 (1)	3 (1.4)	68 (1.5)
Age (y)						
15‐34	141 (21.4)	5 (1.3)	2 (5.1)	140 (4.1)	17 (7.7)	305 (6.5)
35‐54	256 (38.8)	28 (7.3)	7 (17.9)	609 (18)	47(21.2)	947 (20.2)
55‐74	229 (34.7)	263 (68.8)	29 (74.4)	2088 (61.8)	131 (59)	2740 (58.5)
75 or more	25 (3.8)	85 (22.3)	1 (2.6)	527 (15.6)	27 (12.2)	665 (14.2)
No response	8 (1.2)	1 (0.3)	0 (0)	15 (0.4)	0 (0)	24 (0.5)
Gender						
Female	525 (79.7)	243 (63.6)	30 (76.9)	2462 (72.9)	138 (62.2)	3398 (72.6)
Male	114 (17.3)	135 (35.3)	7 (17.9)	881 (26.1)	77 (34.7)	1214 (25.9)
Other/I don’t want to answer or no response	20 (3)	4 (1)	2 (5.1)	36 (1.1)	7 (3.2)	69 (1.5)
Education						
Elementary school or less educated	59 (9)	48 (12.6)	3 (7.7)	354 (10.5)	26 (11.7)	490 (10.5)
12 years school—upper secondary education	213 (32.3)	76 (19.9)	11 (28.2)	854 (25.3)	55 (24.8)	1209 (25.8)
Higher vocational education	103 (15.6)	94 (24.6)	8 (20.5)	783 (23.2)	42 (18.9)	1030 (22)
Higher education ≤3 years	144 (21.9)	73 (19.1)	5 (12.8)	568 (16.8)	29 (13.1)	819 (17.5)
Higher education >3 years	109 (16.5)	69 (18.1)	11 (28.2)	639 (18.9)	47 (21.2)	875 (18.7)
Doctoral education	7 (1.1)	7 (1.8)	0 (0)	62 (1.8)	6 (2.7)	82 (1.8)
Something else or no response	24 (3.6)	15 (3.9)	1 (2.6)	119 (3.5)	17 (7.7)	176 (3.8)
Health care professional education						
Yes	159 (24.1)	73 (19.1)	9 (23.1)	740 (21.9)	34 (15.3)	1015 (21.7)
No	484 (73.4)	296 (77.5)	29 (74.4)	2570 (76.1)	183 (82.4)	3562 (76.1)
No response	16 (2.4)	13 (3.4)	1 (2.6)	69 (2)	5 (2.3)	104 (2.2)
Employment						
Full time	158 (24)	50 (13.1)	7 (17.9)	847 (25.1)	66 (29.7)	1128 (24.1)
Part time	72 (10.9)	18 (4.7)	3 (7.7)	167 (4.9)	18 (8.1)	278 (5.9)
Student	63 (9.6)	2 (0.5)	1 (2.6)	66 (2)	4 (1.8)	136 (2.9)
Retired	181 (27.5)	282 (73.8)	20 (51.3)	2003 (59.3)	115 (51.8)	2601 (55.6)
Not working	148 (22.5)	17 (4.5)	6 (15.4)	189 (5.6)	11 (5)	371 (7.9)
None of the above or no response	37 (5.6)	13 (3.4)	2 (5.1)	107 (3.2)	8 (3.6)	167 (3.6)

### Participant Characteristics

The largest patient group included those who received care for other conditions, representing 72.2% (3379/4681) of the respondents (Table 1). The next largest groups were patients receiving mental health care (659/4681, 14.1%), patients receiving cancer care (382/4681, 8.2%), those who did not receive any care (222/4681, 4.7%), and those receiving care for both mental health and cancer (39/4681, 0.8%).

Overall, the patients tended to be older, with more than half aged 55 years or older. The patients who received mental health care were typically younger, aged 15 to 34 years (141/659, 21.4%), while those who received care for cancer were more likely to be older, aged 75 years or older (85/382, 22.3%). All groups had more female respondents, ranging from 62.2% (138/222) to 79.7% (525/659). All education levels were represented, and more than one-fifth of the patients had health care professional education. Since more than half of the patients were older adults, 55.6% (2601/4681) were retired.

### Statistics and Data Analysis

The statistical data were analyzed using Excel (version 2308; Microsoft Corp) and SPSS (version 29.0.2.0; Lumivero). The statistical analysis was performed using the multivariate binary logistic regression analysis, as the dependent variable was binary and the independent background variables were categorical. The binary logistic regression analysis was conducted in 2 steps. First, model A included variable types of received care based on the hypothesis. In addition, model B included the background variables, such as health condition, age, gender, education, health care professional education, and employment. The employment variable was transformed into 3 categories: combining full time and part time as the working category, student, and combining retired and not working as the outside working life category. Each independent variable used the first category as the reference category. The significance level was set to *P*<.05. The multicollinearity was tested using the Pearson *χ*^2^ test. Each independent variable was tested in pairs with all the others.

Based on the inclusion and exclusion criteria for patient groups, the final sample was 4681, representing 99.2% (4681/4719) of the respondents. In total, 38 (0.8%) responses included conflicting information about the patient group or were missing due to insufficient information about the patient group. In addition, 91 (1.9%; Table 2) respondents did not indicate whether they had felt offended by anything they read in the EHR. Those responses with missing data were excluded, and the final sample size in the quantitative analysis was n=4590, representing 97.3% (4590/4719) of the respondents.

**Table 2.** Frequency of feeling offended among patient groups.

Patient group	Yes, n (%)	No, n (%)	No response, n (%)^a^
Mental health care (n=654)^b^	166 (25.4)	488 (74.6)	5 (0.8)
Cancer care (n=375)^b^	37 (9.9)	338 (90.1)	7 (1.8)
Mental health and cancer care (n=39)^b^	9 (23.1)	30 (76.9)	0 (0)
Other conditions care (n=3316)^b^	383 (11.6)	2933 (88.4)	63 (1.9)
No care (n=206)^b^	22 (10.7)	184 (89.3)	16 (7.2)
Total (n=4590[Table-fn T2_FN2])^b^	617 (13.4)	3973 (86.6)	91 (1.9)

a Percentages have been calculated using the total sample (n=4681), including “No responses.”

b This sample does not include “No responses.”

Since all variables were categorical, a *χ*^2^ test was performed to examine associations between the missingness indicators for felt offended and patient group variables (coded 1=missing value, 0=not missing value) and other variables [58]. For comparisons with the patient group, all variables were nonsignificant except health condition, suggesting the data may be missing completely at random. Those who did not want to share their health condition (2/60, 3.3%) did not respond to the patient group question, differing from other categories (0%‐0.5%) with the significant association (*χ*²_5_=11.54; *P*=.04), suggesting that missingness depends on health conditions and the data may be missing at random. However, the level of missingness is below common thresholds of 5% to 20%, which is typically considered problematic [59].

All variables, except education and patient group, were not significantly associated with the felt offended variable. Among those with no formal education, 10.5% (2/19) did not respond to whether they felt offended, with differences across other categories, with low levels of no responses ranging from 0.9% to 3.0% (*χ*²_7_=15.51; *P*=.03). Respondents across all patient groups had similarly low levels of no responses ranging from 0% to 1.9%, except for those who had not received care in the past 24 months having 7.2% (16/222; *χ*²_5_=39.48; *P*<.001) of no responses. Patients who had not received care may have had less information in their EHR.

### Qualitative Data-Analytic Strategies

The qualitative inductive content analysis was conducted using the ATLAS.ti software (version 24.1.1.30813, ATLAS.ti Scientific Software Development GmbH) and inspired by Harahap et al [60], following their process, including (1) familiarizing authors with data, (2) generating and discussing the codes in collaboration, (3) grouping codes into larger theme categories, (4) evaluating larger theme categories and defining the larger theme categories, and finally presenting larger theme categories and codes with quotes in the *Results* section. Each respondent who felt offended could provide 1 answer to the open-ended question explaining why they felt offended. All responses were analyzed to ensure that inductive thematic saturation was achieved. The same codes repeatedly emerged from the data [61], as agreed by the first and second authors. The responses varied in length from 1 to 131 words, with an average of 10 (SD 11) words.

The first author familiarized themselves with the open-ended responses. Second, they performed preliminary coding using in-vivo coding. In-vivo coding [62] focuses on users’ perspectives rather than predefined options or researchers’ interpretations of the codes. Respondents could provide more than 1 reason for feeling offended, and all reasons were classified under their respective codes. To increase the reliability of the analysis process, the second author discussed and reviewed the codes together with the first author. Third, the first author grouped the codes into larger theme categories. Fourth, the first and second authors collaboratively evaluated and named the larger theme categories. In addition, they created English translations for the codes and larger theme categories. All authors verified the meaning of the codes and larger theme categories.

If both authors were jointly unable to understand the meaning of a response, it was categorized as an “unclear response.” They reviewed together all the unclear responses (39/502, 7.8%). In addition, a few responses (7/502, 1.4%) were classified as “empty response” when respondents explicitly explained that they would not provide additional information, with explanations such as “I don’t want to tell.”

### Ethical Considerations

The study protocol was reviewed and approved by the Aalto University Research Ethics Committee (D/957/03.04/2020 Z) in accordance with the ALLEA European Code of Conduct for Research Integrity and the Finnish Code of Conduct for Research Integrity and Procedures for Handling Alleged Violations of Research Integrity in Finland 2023. The survey invitation included study information and a privacy notice. Responding to the survey was interpreted as indicating that participants had given their informed consent. The informed consent allowed the secondary analysis without additional consent. Participation in the survey was voluntary and anonymous, and no reimbursement was offered. To protect participants’ identities, this study was anonymous and did not include identification variables. If responses included identification information provided by the respondent, it was removed from the data. The data were securely stored in the university’s secure project folder, with restricted access. The identification of individual participants or users in any tables or quotes of the manuscript or supplementary material is not possible.

## Results

### Associations Between Feeling Offended and the Patient Group

The patient group was significantly associated with feeling offended by information. Patients receiving any mental health care (23.1%‐25.4%, Table 2) were more likely than other patients to feel offended by information in their EHRs (9.9%‐11.6%; other care: odds ratio 0.37, 95% CI 0.29‐0.46, *P*<.001, model A; Tables2  3). This statistically significant association persisted even after including additional background variables in model B (Table 3).

Slightly more than every tenth (617/4590, 13.4%) of all respondents reported having felt offended by information in their EHR (Table 2). This level was consistent among cancer care respondents (37/375, 9.9%), respondents with other conditions (383/3316, 11.6%), and those who did not receive care (22/206, 10.7%). Moreover, every fourth (166/654, 25.4%) patient who received mental health care and patients who received both mental health and cancer care (9/39, 23.1%) encountered information they experienced as offensive in their EHR. However, most people (3973/4590, 86.6%) who read their information in their EHR were not offended.

Female patients, students, patients with bad or very bad health condition, those outside working life, or those with a bachelor’s degree or higher were significantly more likely to feel offended by information (Table 3, Multimedia Appendix 2). Older patients were significantly less likely to feel offended by information in their EHRs (Table 3 ). There was no significant difference whether the patient has a health care professional education themselves. The model’s fit was evaluated using Cox and Snell *R²* (0.02 for model A; 0.05 for model B; Multimedia Appendix 3), indicating limited explained variation with a minor improvement in extended model B. The Hosmer–Lemeshow test (*χ*²_1_=0, *P*>.99 for model A; *χ*²_8_=8.67, *P*=.37 for model B) indicated acceptable calibration.

**Table 3.** Multivariate binary logistic regression results—associations between feeling offended, patient group, and background variables.

Model, variable name, and category	Coefficient (B) (SE)	*P* value	Odds ratio (95% CI)
Model A			
Received care (reference: mental health)			
Cancer care	−1.12 (0.21)	<.001	0.33 (0.22‐0.49)
Other care	−1.00 (0.12)	<.001	0.37 (0.29‐0.46)
None	−1.21 (0.28)	<.001	0.30 (0.17‐0.52)
Mental health and cancer care	−0.20 (0.44)	.65	0.82 (0.35‐1.93)
Constant	−1.11 (0.1)	<.001	0.33
Model B			
Received care (reference: mental health)			
Cancer care	−0.70 (0.22)	.002	0.50 (0.32‐0.78)
Other care	−0.55 (0.13)	<.001	0.58 (0.45‐0.75)
None	−0.69 (0.29)	.02	0.50 (0.28‐0.89)
Mental health and cancer care	−0.03 (0.46)	.94	0.97 (0.4‐2.36)
Gender (reference: female)			
Male	−0.35 (0.12)	.005	0.71 (0.56‐0.9)
Age (y) (reference: 15‐34 y old)			
35‐54	0.07 (0.21)	.74	1.07 (0.71‐1.62)
55‐74	−0.53 (0.22)	.02	0.59 (0.38‐0.91)
75 or older	−1.09 (0.28)	<.001	0.34 (0.19‐0.58)
Health care professional (reference: yes)			
No	−0.01 (0.12)	.91	0.99 (0.78‐1.25)
Education (reference: elementary school or no formal education)			
12 years school—upper secondary education	0.38 (0.2)	.06	1.47 (0.99‐2.17)
Higher vocational education (vocational diploma)	0.4 (0.21)	.05	1.5 (1‐2.25)
Higher education ≤3 years (first cycle—bachelor)	0.56 (0.21)	.008	1.75 (1.16‐2.65)
Higher education >3 years (second cycle—master)	0.7 (0.21)	<.001	2.02 (1.35‐3.04)
Research (third cycle) of higher education	0.84 (0.41)	.04	2.32 (1.05‐5.15)
Health condition (reference: very good)			
Good	0.21 (0.31)	.50	1.23 (0.67‐2.25)
Fair	0.46 (0.31)	.14	1.58 (0.87‐2.88)
Bad	1.04 (0.32)	.001	2.82 (1.5‐5.33)
Very bad	1.59 (0.38)	<.001	4.9 (2.35‐10.24)
Employment (reference: working)			
Student	0.59 (0.27)	.03	1.8 (1.06‐3.04)
Outside working life	0.46 (0.13)	<.001	1.58 (1.23‐2.05)
Constant	−2.25 (0.4)	<.001	0.1

### Experiences of Offensive Information in the EHR

Finally, 81.4% (502/617) of respondents who had experience of offense shared more detailed explanation. The respondents were most frequently offended by errors and disrespectful language in their EHRs (Table 4). Patients described instances where their expressions were interpreted wrongly by professionals, or where professionals were not listening during appointments, which was the cause of the error in the records:

*As a patient, what I have said is not written in the appointment’s/hospital care’s records. In addition, the doctor may have interpreted what I said in his own way and in a disrespectful tone*.

[Respondent #3740, Mental health care]

**Table 4.** Inductive content analysis results—types of patient-reported offensive information in the EHR.

Offensive information in the EHR^a^	Frequency, n (%)		
	Total (n=502)	Any mental health care^b^ (n=142)	No mental health care (n=360)
Type of information	197 (39.2)		
Errors	126 (25.1)	26 (18.3)	100 (27.8)
Unnecessary information	60 (12)	25 (17.6)	35 (9.7)
Outdated information	11 (2.2)	4 (2.8)	7 (1.9)
Omissions	8 (1.6)	3 (2.1)	5 (1.4)
Health care professional’s expression in notes	189 (37.6)		
Professional has written disrespectfully	87 (17.3)	20 (14.1)	67 (18.6)
Professional’s opinion/conclusion	42 (8.4)	19 (13.4)	23 (6.4)
Professional’s description of the patient	40 (8)	10 (7)	30 (8.3)
Professional’s word choice	35 (7)	13 (9.2)	22 (6.1)
Information relating to a specific theme	89 (17.7)		
Weight	39 (7.8)	12 (8.5)	27 (7.5)
Use of alcohol or drugs	25 (5)	8 (5.6)	17 (4.7)
Mental health	17 (3.4)	10 (7)	7 (1.9)
Gender	6 (1.2)	1 (0.7)	5 (1.4)
Evaluating the intelligence level	5 (1)	1 (0.7)	4 (1.1)
Communication between patient and professional	51 (10.2)		
Professional has written something that was not discussed in the appointment	19 (3.8)	5 (3.5)	14 (3.9)
Patient has not been heard or believed	19 (3.8)	5 (3.5)	14 (3.9)
Patient disagrees	15 (3)	4 (2.8)	11 (3.1)
Information management	17 (3.4)		
Information shared without patient’s permission	12 (2.4)	3 (2.1)	9 (2.5)
Information was not corrected	5 (1)	0 (0)	5 (1.4)

a EHR: electronic health record.

b Includes individuals who have received both, mental health care and cancer care.

*It offends a bit when you notice that the doctor hasn't listened properly and writes wrong things.*

[Respondent #0300, Other conditions care]

*The doctor’s notes of what happened do not always correspond to my own experience. It is also offensive to skip things and select what ends up being recorded.*

[Respondent #0160, No care]

Moreover, unnecessary information was a common source of offense. Patients described that certain aspects of their lives or personal characteristics were not relevant to their health care:

*In my opinion, the doctor unnecessarily wrote in the notes that me and my spouse no longer have sex.*

[Respondent #0141, Other conditions care]

*How is the treatment relating to if I look cheerful, my former profession, my marital status, etc.*

[Respondent #0201, Other conditions care]

Sometimes, unnecessary information related to certain offensive experience-based topics, for instance, gender:

*As a trans man, I don't understand why ALL records use “woman woman woman” even if gender has nothing to do with it*.

[Respondent #3762, Mental health care]

Additionally, outdated information was a reason for offense. In this example, the offense was related to smoking:

*The doctor added smoking as a risk, I smoked 50 years ago.*

[Respondent #0135, Other conditions care]

The choice of wording or a disrespectful tone in the notes by professionals was also a common source of offense:

*A personal descriptive text written by a nurse when I cried because of cancer. “The patient cries until patient was blue in the face.” Fortunately, the chief medical doctor decided that this was to be removed at my request.*

[Respondent #4367, Cancer care]

The doctor wrote that I am a difficult patient with my difficult pain.

[Respondent #0143, Other conditions care]

Patients described that information relating to a specific theme, such as evaluating intelligence levels or information about weight, was offensive:

*The overweight is mentioned first, and I'm not even fat.*

[Respondent #0078, Other conditions care]

Sometimes, patients described that professionals omitted addressing certain topics, such as weight, during their appointments, even though these topics were documented in the notes:

*The doctor couldn’t directly call me overweight, but I found the text about my information offensive when read. I would have liked the doctor to mention it aloud as well, and not just timidly express it in the text.*

[Respondent #0261, Other conditions care]

Some experiences of being offended were related to communication or the relationship between patients and professionals. Patients described how they have not been heard or believed. By the same token, patients reported disagreements with the professionals:

*The nurse’s diagnosis of my mental state. I disagree with that. I don't like having this kind of note in my records.*

[Respondent #4364, Cancer care]

Concerns were expressed about professionals accessing or sharing the notes without patients’ permission:

*In my opinion, the physiotherapist had looked at information that was not necessary in terms of treatment.*

[Respondent #0057, Other conditions care]

Since patients receiving mental health care appeared to experience offense at a significantly higher rate than other patients, we analyzed and reported their responses separately to deepen our understanding of the factors that could cause this difference.

The reasons for being offended partly differed between those who had received mental health care and those who had not. For the patients who had received mental health care, unnecessary information was related to occasions when mental health was described in the note, even though it was not considered relevant by the respondent when seeking care for somatic problems, as patients expressed here:

*Why is a psychiatric diagnosis always recorded “even if I'm just asking for a plaster on my knee”? How does a psychiatric diagnosis affect wound care?*

[Respondent #3721, Mental health care]

*If I go to the doctor because of a foot problem, the first record I see is a note of recurrent depression*.

[Respondent #3669, Mental health care]

The patients described repeated mentions of their mental health conditions as labeling, as one patient explained:

*No matter what the issue is, it is always remembered to mention that there is a mental health problem. Not all problems are caused by mental health and there is no need to keep reminding about it. It feels like people are being labeled.*

[Respondent #3732, Mental health care]

Additionally, patients mentioned feeling offended when referring to mental health problems that had not been relevant for many years:

*It feels bad to see old mental health diagnoses appearing during a visit, even though they haven't been relevant for years.*

[Respondent #0315, Other conditions care]

Word choice was also a recurrent theme: specific words were mentioned as offensive, and one patient explained how the condition had been described from the patient’s point of view:

*Word choices, eg speaking of a schizophrenic instead of speaking of a person having schizophrenia (a significant difference in tone, although the content is the same*).

[Respondent #3750, Mental health care]

Health care professionals’ opinions, conclusions, and descriptions of patients were often reported as offensive. One patient receiving mental health care described their offense at professionals’ conclusions:

*The doctor considered me mentally unstable due to my respiratory symptoms*.

[Respondent #3701, Mental health care]

### Feeling Offended and Experiences of Offensive Information in the EHR

The patients who had received mental health care, including those who had received care for cancer at the same time, were more likely to feel offended by information in their EHRs. Both groups most frequently reported errors as offensive in their EHR. Among patients who had received mental health care, unnecessary information in their EHR was the second most frequently reported offense, whereas in the other patient group, health care professionals’ disrespectful language was similarly the second most frequently reported offense.

## Discussion

### Principal Findings and Comparison With Prior Work

The patients who had received mental health care, female patients, students, patients with bad or very bad health conditions, those outside working life, or those with a bachelor’s degree or higher were significantly more likely to feel offended by information in their EHR. Patients most frequently experienced errors, health care professionals’ disrespectful language, and unnecessary information as offensive content in their EHRs.

Most patients who read their EHRs did not feel offended, which is consistent with prior work [13,22]. However, patients who had received mental health care were more often offended than other patient groups, as reported in prior findings from Sweden and Norway [25,26]. This may be partly explained by heightened sensitivity to perceived stigma in mental health contexts. The potentially stigmatizing nature of mental health information was also reported in previous research [63,64]. Prior research has shown that individuals with mental health conditions often anticipate or experience negative bias from health care professionals [65]. In this study, patients who received cancer care were not more likely to be offended, which reflects findings from prior qualitative studies [19,23]. However, our study shows that patients who have received both cancer and mental health care are more likely to feel offended.

The findings that females and patients with bad or very bad health conditions were more likely to feel offended are in accordance with Fernández et al [22]. Those offensive experiences with the EHR may reflect other care as well. For example, Silva et al [66] have described how the EHR may negatively affect women’s relationships with health care professionals and attitudes toward health care. On the other hand, older patients were less likely to feel offended by information. This is consistent with previous findings of Hagström et al [67], where more than a quarter of adolescents and young adults had been offended by reading their EHR.

This study found that some patients experienced certain information in their EHR as offensive. Errors, health care professionals’ disrespectful language, and unnecessary information were the most frequently mentioned reasons for being offended. These findings suggest that both accuracy and a respectful tone are central to how patients evaluate clinical documentation. These results match those observed in earlier studies in the United States [22,33,63,68,69]. Another important finding is that patients who received mental health care more frequently reported unnecessary information and professionals’ opinions, conclusions, and word choices as offensive compared to those who had not received mental health care. One reason could be patients’ heightened sensitivity to language and labeling. Evaluating the intelligence level, professionals’ opinions and conclusions, information that was not corrected, and outdated information have not been pointed out in previous studies as reasons for feeling offended. Even though information can be medically essential, it should be formulated politely, and health care professionals should receive support in their writing practices.

In accordance with these results, previous studies have observed that patients take offense if the information is not consistent with the message that was verbally conveyed during the appointment [12,22] or if the patient disagrees and is not heard [22]. Thus, a better understanding of medical information from the patient’s point of view can also determine the relationship between patients and professionals. In a previous study, the patient not being heard was even described as a “blind spot” that might affect other aspects, such as patient safety or quality of care [70].

### Practical Implications

Based on these findings, we formulated guidelines to support the development of EHRs. First, information in EHRs should be coherent. Errors and omissions might be caused by technical issues or the health care professional’s writing process. Technical coherence in processing the information should be guaranteed. In addition, patients should be able to report potential errors and omissions to professionals because uncorrected records were explicitly identified as offensive by some respondents, and patients face challenges in correcting errors in their EHRs [8,24].

Second, health care professionals should tell patients why certain information is relevant. Patients, particularly those with mental health conditions, could be informed about how some outdated or unnecessary information might be important from a medical point of view, even though it would be considered old or irrelevant by the patient. These patients interpreted such mentions as labeling or stigmatizing, even when the information may have had medical value. Additionally, patients should have the option to limit the sharing of their information or receive notifications if their information needs to be shared.

Third, health care professionals should receive education on how to highlight patients’ perspectives in their records and receive recommendations on appropriate descriptions of patients, their reactions, and word choices to avoid language that patients perceive as offensive. Moreover, technical tools, such as word lists or standard phrases, could support or remind professionals when writing records. The respondents’ examples of differences in tone, such as using “a person having schizophrenia” instead of “a schizophrenic,” describe how patient feedback could be used to guide health care professionals. Volkow et al have suggested using that person-centered language, reducing the stigma around mental health conditions [71]. Word lists and standard phrases should be created by involving patients, caregivers, and professionals in the planning process to ensure that they reduce the risk of offense when patients have access to their EHR.

Fourth, EHRs can influence communication between patients and health care professionals. Even though certain information might not be medically relevant to the current situation, it could be important for the patient. Additionally, professionals could explain their conclusions to patients, especially those with mental health conditions, as these might otherwise be considered offensive. This could support patients’ understanding of their health information, which may be difficult to comprehend [8,24,72].

### Limitations and Future Directions

The generalizability of these results has certain limitations. This study is based on a relatively small sample of participants. It was limited to one patient portal, My Kanta, in Finland, with a short 3-week period and a low response rate of 0.37% (4719/1,262,708). The response rate is, however, consistent with other similar surveys, such as in Sweden, which had a response rate of 0.61% (2587/423,141) [73], and a previous research in Finland, which reported a response rate of 0.7% (3139/449,922) [72]. Although there were more older adults and females among the respondents, they represent the most active users of the Finnish national patient portal [74]. Additionally, they align with the respondents from a previous study on the Finnish national patient portal for pharmacy customers [75]. Since the survey was available for only 3 weeks via the patient portal, frequent users may be overrepresented. The patient groups displayed variability in participant demographics, making the creation of unbiased subgroups inapplicable. However, the variability facilitated reaching different groups.

Even though this survey was offered to all national patient portal users, the language options were limited to the official languages of Finland: Finnish and Swedish. Thus, this study had limited data of those who are not able to use Finnish or Swedish. Future research is needed to explore how nonnative patient groups may experience the content of the EHR and whether experiences of offense vary.

Second, the contexts of language, expectations, and EHR writing practices may vary by culture and country. Future research could usefully explore how cultural variations may affect patients’ experiences. However, the attitudes and stigma surrounding mental health conditions have been widely identified [71]. One limitation of the study was that the concept of offense was not defined, and it was measured with a single-item question. In addition, the survey as a research method has limited opportunities to request more detailed information from the respondents. On the other hand, the survey provided anonymity, making it easier for respondents to describe challenging topics related to offensive health information in their own words. Even though the responses were shorter than the interview would allow, we were able to collect experiences from a large sample of patients using the survey. Future research using interviews or focus groups would enhance a deeper understanding of these findings.

This study focused on patients’ experiences. Since errors in EHRs were the most frequently mentioned reason for being offended, further work is required to thoroughly understand the nature and severity of these errors. Future research is also needed to explore whether experiences of offense had an impact on trust in care progress and the relationship between patients and professionals. Moreover, further studies that consider clinical outcomes and health care professionals’ perspectives could be undertaken.

### Conclusions

The purpose of this study was to explore whether certain patient groups are more likely to feel offended while reading their EHRs, the types of information that patients find offensive, and to provide a comparison across multiple patient groups using a mixed methods approach. Prior studies have often focused on single patient groups or specific clinical contexts, leaving a limited understanding of differences across multiple patient groups. This study contributes new knowledge by identifying differences across multiple patient groups. The results highlight differences in experiences of offense between those who have received mental health care and those who have not. Health care professionals could consider that some patients might feel offended by certain types of information identified in this study or tone of expressions in their EHR. This is particularly important for patients with mental health conditions because they are more likely to be offended by reading their EHR.

Health care professionals could be provided with education that includes this information, enabling them to use that knowledge as part of the process. Improving the quality of EHRs could strengthen the relationship between patients and professionals. Furthermore, to support communication between professionals and patients, patients could receive information about why certain specific themes are necessary to record, even though they might be sensitive topics and feel unnecessary in that context.

## Supplementary material

10.2196/86178 Multimedia Appendix 1 The survey questions in English.

10.2196/86178 Multimedia Appendix 2 Felt offended and background variables table.

10.2196/86178 Multimedia Appendix 3 Evaluation of models fit table.

10.2196/86178 Checklist 1 CHERRIES checklist.

## Abbreviations

CHERRIES: Checklist for Reporting Results of Internet E-Surveys
EHR: electronic health record
