# Supplementary material for: Understanding Patient-Reported Offenses in Electronic Health Records: Cross-Sectional Mixed Methods Survey
Source: J Med Internet Res. 2026 May 14;28:e86178. doi: 10.2196/86178 (PMC13175307; doi:10.2196/86178)
Supplement: Multimedia Appendix 1 [file jmir-v28-e86178-s001.pdf]

Multimedia Appendix 2

The survey questions in English.

Have you ever felt offended by something you read?

Yes/ No

If YES, please explain.

How is your overall health?

Very good/ Good/ Fair/ Bad/ Very bad/ I don't know / I don't want to answer

In the last 2 years, have you received care from a doctor (GP or specialist) or other health professional for [select all that apply]

Mental health condition(s)/ Cancer/ Other health problem(s)/ No treatment

Gender

Female/ Male/ Other

Age

15 to 19 years old/ 20 to 24 years old/ 25 to 34 years old/ 35 to 44 years old/ 45 to 54 years old/ 55 to 64 years old/ 65 to 74 years old/ 75 to 84 years old/ 85 years old or older

Highest completed education

No formal education/ Elementary school/12 years school - Upper secondary education/ Higher vocational education/ Higher education ≤ 3 years/ Higher education, >3 years/ Doctoral education

Do you have a health care professional education?

Yes/ No

Which of the following best describes your current employment status? Choose the one that is most relevant.

Full time/ Part time/ Student/ Retired/ Unemployed/ Not able to work/ None of the above (free text)
