# Supplementary material for: Understanding Patient-Reported Offenses in Electronic Health Records: Cross-Sectional Mixed Methods Survey
Source: J Med Internet Res. 2026 May 14;28:e86178. doi: 10.2196/86178 (PMC13175307; doi:10.2196/86178)
Supplement: Multimedia Appendix 2 [file jmir-v28-e86178-s002.pdf]

Multimedia Appendix 3

Felt offended and background variables table.

Table. Felt offended and background parameters per patient group.

Patient group		Mental health, n=659	Cancer, n=382	Mental health & cancer, n=39	Other conditions, n=3379	No care, n=222	Total, N=4681
		Felt offended, Yes	Felt offended, Yes	Felt offended, Yes	Felt offended, Yes	Felt offended, Yes	Felt offended, Yes
**Health condition, n (%)**							
	Very good	2 (18.2)	1 (20)	0 (0)	14 (10.9)	1 (3.3)	18 (10.2)
	Good	33 (22.9)	11 (10.8)	1 (10)	109 (9.2)	7 (7.3)	161 (10.5)
	Fair	69 (22.6)	12 (6.3)	2 (15.4)	175 (10.9)	5 (8.5)	263 (12.1)
	Bad	44 (32.8)	8 (15.1)	6 (50)	56 (17.7)	4 (28.6)	118 (22.3)
	Very bad	14 (36.8)	3 (18.8)	0 (0)	18 (35.3)	4 (80)	39 (35.1)
**Age, n (%)**							
	15 – 34 years	46 (32.6)	1 (20)	0 (0)	20 (14.5)	2 (11.8)	69 (22.8)
	35 – 54 years	72 (28.2)	5 (18.5)	4 (57.1)	98 (16.6)	4 (9.3)	183 (19.9)
	55 – 74 years	45 (19.8)	27 (10.4)	5 (17.2)	226 (11)	14 (11.7)	317 (11.8)
	75 years or more	1 (4.2)	4 (4.8)	0 (0)	39 (7.5)	2 (7.7)	46 (7.1)
**Gender, n (%)**							
	Female	126 (24.2)	27 (11.3)	7 (23.3)	301 (12.4)	11 (8.7)	472 (14.2)
	Male	31 (27.2)	8 (6.1)	1 (14.3)	65 (7.5)	8 (11.1)	113 (9.5)
**Education, n (%)**							
	Elementary school or less educated	11 (19)	3 (6.5)	2 (66.7)	23 (6.6)	2 (7.7)	41 (8.5)
	12 years school - Upper secondary education	55 (25.9)	6 (7.9)	2 (18.2)	97 (11.5)	2 (3.8)	162 (13.6)
	Higher vocational education	24 (23.8)	12 (13.2)	0 (0)	75 (9.7)	5 (13.9)	116 (11.5)

	Higher education ≤ 3 years	43 (30.1)	5 (6.9)	2 (40)	71 (13)	1 (3.6)	122 (15.4)
	Higher education, >3 years	23 (21.1)	11 (16.2)	3 (27.3)	92 (14.6)	10 (24.4)	139 (16.2)
	Doctoral education	1 (14.3)	0 (0)	-	9 (15)	1 (16.7)	11 (13.8)
**Health care professional education, n (%)**							
	Yes	35 (22.3)	9 (12.3)	1 (11.1)	90 (12.4)	4 (13.3)	139 (14)
	No	126 (26.2)	27 (9.3)	7 (24.1)	284 (11.3)	17 (9.9)	461 (13.2)
**Employment, n (%)**							
	Working	52 (22.6)	7 (10.3)	3 (30)	96 (9.5)	7 (8.3)	165 (11.7)
	Student	18 (28.6)	1 (50)	0 (0)	16 (25.8)	1 (25)	36 (27.3)
	Not working	85 (25.8)	27 (9)	5 (19.2)	242 (11)	11 (8.7)	370 (12.4)
