# Supplementary material for: Understanding Patient-Reported Offenses in Electronic Health Records: Cross-Sectional Mixed Methods Survey
Source: J Med Internet Res. 2026 May 14;28:e86178. doi: 10.2196/86178 (PMC13175307; doi:10.2196/86178)
Supplement: Multimedia Appendix 3 [file jmir-v28-e86178-s003.pdf]

Multimedia Appendix 4

Evaluation of models fit table.

Evaluation of models fit in multivariate binary logistic regression analysis.

Model	Model A	Model B
χ^2	76.84	196.79
df	4	20
P value	<.001	<.001
χ^2 (Hosmer-Lemeshow)	0.000	8.67
df (Hosmer-Lemeshow)	1	8
P value (Hosmer-Lemeshow)	1.000	.37
-2 Log Likelihood	3028.15	2908.2
Cox & Snell R^2	0.02	0.05
Nagelkerke R^2	0.04	0.09
Variables	Received Care	Received Care, Age, Gender, Health Care Professional Education, Education, Health Condition, Employment
